# Collaborative Team Training in Virtual Reality is Superior to Individual Learning For Performing Complex Open Surgery: A Randomized Controlled Trial

**DOI:** 10.1097/SLA.0000000000006079

**Published:** 2023-08-28

**Authors:** Thomas C. Edwards, Daniella Soussi, Shubham Gupta, Sikandar Khan, Arjun Patel, Amogh Patil, Alexander D. Liddle, Justin P. Cobb, Kartik Logishetty

**Affiliations:** MSk Lab, Imperial College London, London, UK

**Keywords:** interprofessional education, patient safety, simulation, surgical teams, virtual reality

## Abstract

**Objective:**

To assess whether multiplayer immersive Virtual Reality (iVR) training was superior to single-player training for the acquisition of both technical and nontechnical skills in learning complex surgery.

**Background:**

Superior teamwork in the operating room (OR) is associated with improved technical performance and clinical outcomes. iVR can successfully train OR staff individually; however, iVR team training has yet to be investigated.

**Methods:**

Forty participants were randomized to individual or team iVR training. Individually trained participants practiced alongside virtual avatar counterparts, whereas teams trained live in pairs. Both groups underwent 5 iVR training sessions over 6 weeks. Subsequently, they completed a real-life assessment in which they performed anterior approach total hip arthroplasty surgery on a high-fidelity model with real equipment in a simulated OR. Teams performed together, and individually trained participants were randomly paired up. Videos were marked by 2 blinded assessors recording the ‘Non-Operative Technical Skills for Surgeons, Oxford NOn-TECHnical Skills II and Scrub Practitioners’ List of Intraoperative Non-Technical Skills’ scores. Secondary outcomes were procedure duration and the number of technical errors.

**Results:**

Teams outperformed individually trained participants for nontechnical skills in the real-world assessment (Non-Operative Technical Skills for Surgeons: 13.1±1.5 vs 10.6±1.6, *P* = 0.002, Non-TECHnical Skills II score: 51.7 ± 5.5 vs 42.3 ± 5.6, *P* = 0.001 and Scrub Practitioners’ List of Intraoperative Non-Technical Skills: 10 ± 1.2 vs 7.9 ± 1.6, *P* = 0.004). They completed the assessment 33% faster (28.2 minutes ± 5.5 vs 41.8 ± 8.9, *P* < 0.001), and made fewer than half the number of technical errors (10.4 ± 6.1 vs 22.6 ± 5.4, *P* < 0.001).

**Conclusions:**

Multiplayer training leads to faster surgery with fewer technical errors and the development of superior nontechnical skills.

Adverse events occur in around 1 in 10 admissions to hospital.^1–3^ The most common place for these to happen is in the operating room (OR), with the majority of these surgical errors considered preventable.^1,4–6^ To improve patient safety, a number of measures have been suggested to reduce the incidence of error, focussing on both individual roles and team performance.^7–9^

The surgical team is comprised of a surgeon, anesthesiologist, scrub technician/nurse, anesthetic assistant, and circulating staff. Superior team performance is strongly associated with a reduction in adverse events, complications, and mortality, and with improved patient outcomes.^10,11^ It also has an indirect effect on outcomes by promoting greater surgical efficiency and shorter operative times, which in themselves result in a reduced chance of a serious complication occurring.^12^ Therefore, highly performing surgical teams who deliver efficient operations reduce the likelihood of patients coming to harm.

Implementation of interventions that focus on team performance within surgery has resulted in both improvements in nontechnical skills and also, reduced intraoperative technical error.^7^ However, although these interventions, which are based on crew-resource management (CRM) training within the aviation industry, are effective, they are often delivered in course format, requiring significant resources and time off work for participants.

Immersive virtual reality (iVR), is an easily accessible technology where participants from anywhere in the world can enter a virtual OR and perform surgery using a motion-tracked headset and controllers.^13^ It has proven effective in training junior surgeons to perform both endoscopic and complex open procedures.^13,14^ Similarly, scrub technicians, who in many parts of the world have limited structured training, have been shown to benefit from a virtual reality curriculum in complex revision total knee arthroplasty surgery.^15^ However, despite lending itself perfectly to collaborative learning in the virtual world, iVR has yet to be used for multidisciplinary team training in the OR.

The anterior approach (AA) for total hip arthroplasty (THA) is known to be technically challenging with a strenuous learning curve.^16^ Complication rates of up to 20% have been reported for surgeons learning this operation, reducing to 7% once accomplished.^17^ This study aimed to investigate whether an innovative collaborative team iVR module was superior when compared with conventional single-player iVR training. As a technically difficult, multistep open operation, the AA-THA was chosen to test the hypothesis that this collaborative approach will be superior to individual learning.

## METHODS

### Setting and Participants

The study protocol was registered (ISRCTN32225943) and ethical approval was granted prospectively by the Health Research Authority (REC reference: 18/HRA/2085, IRAS ID: 237607). This research was conducted in a specially designed iVR training facility, in the simulation laboratory at Imperial College, London. Between April and October 2021, participants were recruited for one of 2 roles: surgeon or scrub technician. Junior orthopedic surgical residents in their second to fifth year post qualification (foundation year 2 to specialist trainee year 3 in the UK terminology) were eligible to be recruited for the surgeon role. They were excluded if they had previously performed any supervised AA-THA operations, >10 THA operations by any approach, had previous participation in orthopedic surgery iVR simulation, or if they could not commit the required time to the study. Undergraduate nursing students, medical students, and qualified scrub technicians or anesthetic assistants, within their first year of training, were eligible to be recruited for the scrub technician role. Participants were excluded if they had previous experience scrubbing for AA-THA procedures, >1-year experience in an orthopedic scrub role of any kind, prior training in THA instrumentation, previous orthopedic iVR simulation experience, or if they were unable to commit the required time to the study. All participants provided written informed consent to participate.

### Randomization

Participants were randomized to one of 2 parallel groups using a block randomization protocol in a 1:1 allocation ratio using an online computer-generated random number sequence by a physician associate not involved in the trial. Participants were randomized to either solo iVR (with the individual training as either a surgeon or scrub technician) or team iVR (training with a co-participant surgeon or scrub technician). Group allocation was concealed until participants were fully enrolled in the study.

### Baseline Visit

At baseline, all participants provided demographic information and underwent a short written, role-specific baseline knowledge assessment. This was developed to assess key skills and knowledge required to perform the operation in the real world. For surgeon participants, this involved instrumentation terminology and application, procedural steps, and understanding of the target orientation of components and technique (14 questions). Scrub technicians were assessed on their knowledge of instrumentation, procedural sequence, and a practical element asking them to assemble equipment (19 questions). A short introductory presentation was subsequently delivered to all participants to standardize baseline knowledge.

### Immersive Virtual Reality Training

**FIGURE 1:**
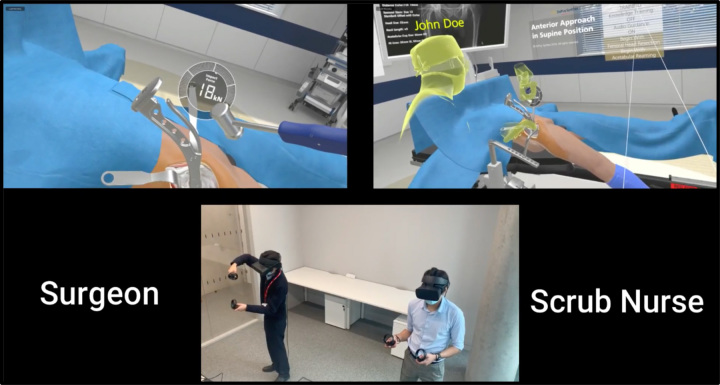
The equipment set up for the multiplayer mode. The images in the top right and left demonstrate the view through the headsets for the surgeon (right) and scrub technician (left). The image below shows the hardware (headset and motion-tracked controllers) being used in a team training session.

The software used in this study was a bespoke team training package created through the augmentation of a preexisting and validated AA-THA module (Pixelmolkerei, Chur, Switzerland).^13^ This module, a previous AA-THA cognitive task analysis,^18^ and intraoperative video footage were interrogated to divide the choreography into key steps for surgeons and scrub technicians. Once created, the module was beta-tested with iterative feedback from experienced scrub technicians, surgeons, and lay representatives to further refine the system. Three modes were established: multiplayer (scrub technician and surgeon training live in pairs), solo scrub technician, and solo surgeon (training with a computer avatar playing the alternative role). In the solo mode, participants completed the steps for their role; once a step was fully complete, the computer avatar would respond by moving on to perform the next task in their sequence. Solo participants were not able to verbally interact with the avatar. The training was otherwise identical between the multiplayer and solo modes, teaching them to perform an AA-THA in the supine position. It guides participants through their role-specific tasks with audio commentary, identifying the equipment needed at each stage and illustrating how to complete the key steps. This was delivered using an Oculus Rift S headset and 2 hand-held motion-tracked controllers (Meta Platforms; Fig. 1). Each training session lasted ~90 minutes with 30 minutes for training, followed by 60 minutes of assessment and was supervised by an iVR technician who provided technical support and ensured the safety of the participants. In the assessment mode, participants were not guided; however automatic, computer-generated prompts were provided when progress was not made after 30 seconds. All participants underwent 5 iVR sessions over the 6-week period. This timetable was chosen as previous studies have indicated that the learning curve in iVR surgery training reaches a plateau after 4 sessions.^19^

### Real-world Assessment

**FIGURE 2:**
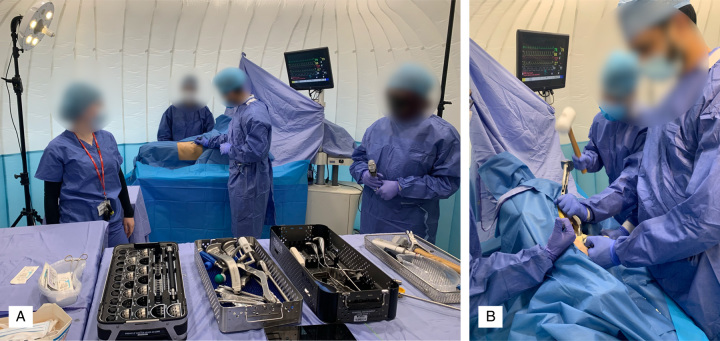
The setup for the real-world assessment using the distributed simulation. A, Demonstrates the overall setup with equipment, personnel, and model. B, Shows a participant broaching the femur during the simulated operation.

After the final iVR session, participants completed a real-world assessment on a high-fidelity model with silicone skin, subcutaneous fat, fascia, capsule and a validated saw bone femur and pelvis (Sawbones, Pacific Research Laboratories). The assessment took place in a 360-degree distributed simulation operating room (Fig. 2). This setup has been validated as an appropriate medium to test both technical and nontechnical skills.^20^ Team-trained participants performed the assessment in their training pairs, solo participants were randomly paired, using a computer-generated random number sequence, with another solo participant of the opposite role. Participants performed the full procedure wearing a surgical gown, gloves, and cap using real surgical instruments (Fig. 2). They were instructed to perform the procedure together exactly as they had been taught in iVR. Participants were assisted by 3 passive surgical assistants; 2 of whom held retractors as directed and a third who operated the traction table for manipulation of the femur. They were only prompted if they requested help or they were performing an unsafe action or one which may jeopardize the remainder of the assessment. The assessment was filmed using 3 cameras (GoPro HERO7, GoPro) stationed around the operating room enabling assessment of the surgeon, the scrub technician, and their teamwork.

### Video Assessment—Nontechnical skills

Video recordings of assessments were analyzed independently by 2 assessors who were not involved in the iVR training and were blinded to the participant group. One assessor was a senior hip arthroplasty fellow with over 10 years of postgraduate experience, the other was an orthopedic surgery trainee with 4 years of postgraduate experience. Both had specific training using 3 nontechnical skills scores: (1) Non-Operative Technical Skills for Surgeons (NOTSS) (primary outcome measure), (2) the Oxford NOn-TECHnical Skills II (NOTECHS-II) score, and (3) the Scrub Practitioners’ List of Intraoperative Non-Technical Skills (SPLINTS) score. All 3 scores have been validated and have been demonstrated to be reliable and reproducible.^21–23^ The mean scores were used and interobserver reliability was calculated using the intraclass correlation coefficient. Details of the scoring methodology for each metric are published elsewhere.^21–23^ In short, each score grades participants’ performance in several well-established nontechnical subdomains. These include: situation awareness (SA), teamwork and communication (T&C), decision-making (DM), problem-solving (PS), leadership (L), and task management. The NOTSS score focuses on the surgeon, grading each of 4 subdomains (SA, T&C, DM, and L) out of 4. These scores are subsequently added together with the highest possible score (indicating the best nontechnical performance) being 16 and the lowest score being 4.^21^ The SPLINTS score takes a similar approach focusing on the scrub technician, grading 3 subdomains (SA, T&C, and task management) out of 4, giving a maximum score of 12.^23^ The NOTECHS-II score examines the nontechnical performance of each surgical team participant individually (surgeon, scrub technician, and anesthetist), before adding the scores together. The 4 subdomains (L, PS, DM and T&C, and SA) are graded out of 8 providing a maximum score for each participant of 32. The maximum possible score for all 3 participants would be 96; however, this was adapted in the present study to include just the scrub technician and surgeon with the best possible score being 64.^22^

### Video Assessment—Technical Skills

Technical skills were assessed by the 2 blinded observers independently using the same assessment video footage. An 80-point task-specific checklist for the AA-THA (Supplemental Digital Content Table 2, http://links.lww.com/SLA/E852) was created from the previously expert-derived and validated AA-THA module (Pixelmolkerei, Chur, Switzerland).^13,19^ The steps from this checklist were used to assess the real-world assessment. Surgical teams were graded on the number of steps from the task-specific checklist they successfully completed. Procedural errors were calculated by subtracting the number of successfully completed steps from the maximum possible score of 80.

### Acetabular Component Orientation

Accurate acetabular component orientation is a well-established surrogate of technical proficiency in hip arthroplasty, with mal-positioning being closely associated with complications, such as dislocation, impingement, accelerated polyethylene wear, and revision surgery.^24–26^ Furthermore, in AA-THA there is a greater propensity to place this component outside the target safe zone reported when compared with other approaches.^27^ As such, surgeons were assessed for their acetabular component positioning using a digital goniometer (Digital Angle Gauge, Wixey, USA). This was measured according to their error in degrees from the prescribed target of 20 degrees anteversion and 40 degrees of inclination, selected to be well within the widely accepted ‘safe zone’.^26,28,29^ Anteversion was measured in relation to the anterior pelvic plane, which was made parallel to the operating table for the assessment performed in the supine position. The digital goniometer was calibrated at zero degrees on the table and placed horizontally on the introducer. Acetabular inclination was measured in relation to the axial plane of the pelvis. To measure it, the pelvis was rotated 90 degrees from the supine position and then the same process was repeated.

### Sample Size

An a priori calculation for sample size was made for the NOTSS score as the primary outcome measure. The minimum effect size was calculated from a comparable simulation study by Brunckhorst et al,^20^ who measured surgical trainees’ NOTSS scores in a similar distributed simulation environment. This article determined an effect size of 1.34 SDs between control and intervention groups (total NOTSS scores, mean ± SD: control: 9.1 0 ±3.42, intervention: 13.1 ± 2.49). To achieve the power of 80% with an alpha of 0.05, 40 participants (20 for each arm, 10 teams, and 10 solo pairs for the final real-world assessment) were required.

### Statistical Analysis

Statistical analysis was performed using Stata (Stata/IC 10.1, StataCorp LP). Interobserver reliability between the two video analyzers was assessed using a 2-way, intraclass correlation coefficient, where a score above 0.75 generally indicates good agreement.^30^ Data comparing group performances were tested for normality using the Shapiro-Wilk test alongside visualization of the data through histograms. Variables with normal distribution were analyzed utilizing the independent samples *t* test. Nonparametric variables were analyzed using the Mann-Whitney *U* Test. A 2-sided *P* value of ≤0.05 was deemed statistically significant. All results are stated as mean ± SD unless stated otherwise.

## RESULTS

Forty-six participants were initially screened for eligibility, 6 declined to participate due to the time commitment. Forty subjects fully enrolled in the study as shown in the CONSORT flow diagram. The demographics of the participants are shown in Tables 1 and 2. There were no significant differences between groups comparing baseline knowledge scores for surgeons (team 46.5% ± 11.9% vs 34.3% ± 17.1%, *P* = 0.08) or scrub technicians (team 46.9% ± 28.4% vs 50.5% ± 27.9%, *P* = 0.776)

**TABLE 1:** Summary of Demographics for Surgical Residents

Characteristic	Team surgeons (n = 10)	Solo surgeons (n = 10)	*P*
Sex (M:F) (n)	7:3	10:0	0.211
Mean age (yr±SD)	29.2±3.0	28.5±2.1	0.552^*^
Hand dominance (right:left)	9:1	10:0	1.00
Baseline knowledge score (%±SD)	46.5±11.9	34.3±17.1	0.08^*^
Level of training			0.243
FY2	1	0	—
CT1	3	7	—
CT2	5	2	—
ST3	1	1	—
Video game previous experience			0.103
Never	0	0	—
Rarely	4	0	—
Occasionally	4	4	—
Frequently	2	5	—
Very frequently	0	1	—
iVR experience			0.591
Never	4	2	—
Rarely	4	3	—
Occasionally	2	3	—
Frequently	0	2	—
Very frequently	0	0	—

* Independent samples students *t* test, otherwise Fisher exact test (categorical data).

CT1 indicates core trainee year 1; CT2, core trainee year 2; FY2, foundation year 2; ST3, specialist trainee year 3.

**TABLE 2:** Summary of Demographics for Scrub Technicians

Characteristic	Team scrub (n = 10)	Solo scrub (n = 10)	*P*
Sex			1.00
Male	3	3	—
Female	6	7	—
Nonbinary	1	0	—
Mean age (yr±SD)	24±6.1	24.7±6.3	0.803^*^
Hand dominance (right:left)	10:0	9:1	1.00
Baseline knowledge score (%±SD)	46.9±28.4	50.5±27.9	0.776^*^
Role			
Nursing student	2	3	1.00
Medical student	6	6	—
Junior scrub technician	1	1	—
Anesthetic assistant	1	0	—
Video game previous experience			0.039
Never	3	0	—
Rarely	0	4	—
Occasionally	6	4	—
Frequently	1	1	—
Very frequently	0	1	—
iVR experience			0.656
Never	6	4	—
Rarely	4	5	—
Occasionally	0	0	—
Frequently	0	1	—
Very frequently	0	0	—

* Independent samples students *t* test, otherwise Fisher exact test used (categorical data).

### Nontechnical Performance

**FIGURE 3:**
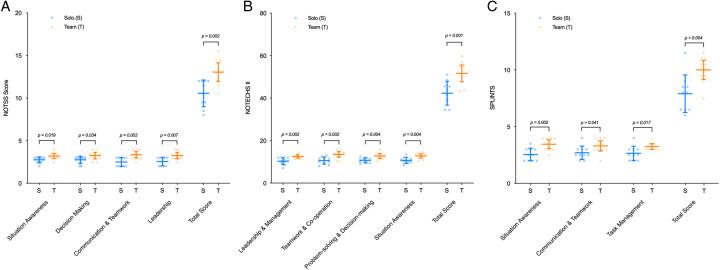
Column scatter plots demonstrating the nontechnical performance in the real-world assessment for the 3 measured scores: (A) NOTSS (B) NOTECHS-II, and (C) SPLINTS, for team (T) and solo (S) trained participants. The central horizontal line within the box shows the mean. The whiskers demonstrate the SD. Significant *P* values (<0.05) are indicated.

Table 3 summarizes the study’s key findings. For nontechnical performance, team-trained participants outperformed the solo groups in all of the 3 nontechnical elements. NOTSS (13.1 ± 1.5 vs 10.6 ± 1.6, *P* = 0.002), NOTECHS-II (51.7± 5.5 vs 42.3 ± 5.6, *P* = 0.001), and SPLINTS (10 ± 1.2 vs 7.9 ± 1.6, *P* = 0.004) (Fig. 3).

### Technical Performance

**FIGURE 4:**
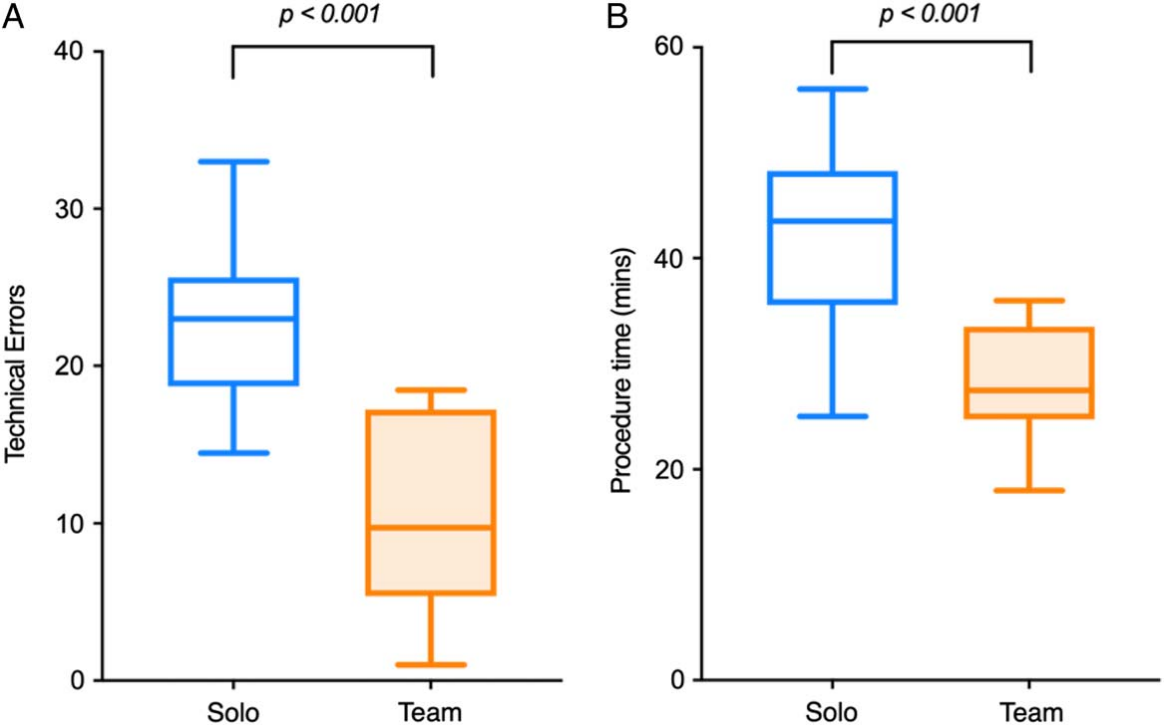
Column scatter plots demonstrating the technical performance metrics in the real-world assessment: (A) technical error count and (B) procedure duration in minutes, for team (T) and solo (S) trained participants. The central horizontal line within the box shows the mean. The whiskers demonstrate the SD. Significant *P* values (<0.05) are indicated.

Team-trained participants performed the procedure 33% faster when compared with the solo group (28.2 minutes ±5.5 vs 41.8 ± 8.9, *P* < 0.001) and made fewer than half the number of procedural errors (10.4 ± 6.1 vs 22.6±5.4, *P* < 0.001) (Fig. 4). Supplemental Table (Supplemental Digital Content Table 2, http://links.lww.com/SLA/E852) demonstrates the detailed breakdown of errors made between team and solo participants. There were no significant differences in the accuracy of acetabular component orientation measurements between groups (Table 3).

**TABLE 3:** Summary of Nontechnical and Technical Outcomes in the Real-world Assessment

Variable	Team (n = 10)	Solo (n = 10)	*P*
NOTSS	13.1±1.5	10.6±1.6	0.002^*^
NOTECHS-II	51.7±5.5	42.3±5.6	0.001^*^
SPLINTS	10±1.2	7.9±1.6	0.004^*^
Procedural errors	10.4±6.1	22.6±5.4	<0.001^*^
Anteversion error (degree)	5.9±4.3	6.9±4.8	0.606
Inclination error (degree)	5.7±4.5	4.9±3.3	0.680
Procedure time (min)	28.2±5.5	41.8±8.9	<0.001^*^

* Statistically significant result.

Data are presented as mean ± SD; means compared using the independent samples student *t* test.

### Reliability

Interobserver reliability was excellent for all video measured metrics; NOTSS: 0.92 (95% CI: 0.79–0.97), NOTECHS-II: 0.94 (95% CI: 0.84–0.98), SPLINTS: 0.92 (95% CI: 0.80–0.97), and technical errors: 0.99 (95% CI: 0.98–1.00); Supplemental Table (Supplemental Digital Content Table 1, http://links.lww.com/SLA/E852).

## DISCUSSION

The most important finding of this study was that those who trained in a team exhibited superior nontechnical skills, performing the operations more efficiently, and with fewer technical errors when compared with those who trained alone. The use of iVR simulation to facilitate the delivery of this training seems to be feasible and highly effective.

The association between good teamwork skills and improved patient safety is now well established.^9–11,31^ One of the key findings of the present study was that the superior nontechnical skills exhibited by team-trained participants were associated with a reduced number of procedural errors. Several other authors have supported these findings, linking superior nontechnical performance to reduced surgical error, complications, mortality, and improved outcomes.^10,11,31^ Fecso et al^10^ focused on technical adverse events in bariatric surgery, the authors noted superior nontechnical performance for both scrub technicians and surgeons to be linked to a reduction in technical adverse events. Similarly, Mazzocco and colleagues, in a study of 300 observed surgeries, suggested that mortality and significant complications were more likely when a paucity of good intraoperative teamwork behavior was observed.^11^ The work by Catchpole and colleagues concurs with the findings of the present study, examining technical errors and nontechnical skills for surgical teams performing 2 common general surgical procedures.^31^ The authors report that superior scores in both nursing and surgical domains of the NOTECHS-II were correlated with a decreased chance of observing a technical error. They conclude that interventions designed to improve teamwork may be beneficial in terms of technical error and patient outcomes. It is worth noting that these studies were conducted in the clinical environment, whereas the present study was assessed in a simulation. The advantage of our study is the reduction in potential bias through its randomized design. The similar conclusions drawn give support to the notion that the benefits seen through team training in the simulation may transfer to the physical world. This has important patient safety implications through improving technical proficiency and error reduction, which are both linked to reduced complication rates and superior patient outcomes.^32^

A second benefit of the team training was increased efficiency, with a 33% reduction in overall procedure time recorded. This may also have a beneficial effect on patient safety; there is now a substantial body of evidence linking prolonged operation times to an increased risk of developing significant complications.^12,33,34^ In a recent registry-based study including 92,343 total knee arthroplasty operations, surgical duration >100 minutes were associated with almost double the risk of experiencing deep infection.^12^ Similar findings have been demonstrated by other authors highlighting considerable reductions in complications with shorter, more efficient operations.^33,34^ The presented evidence would suggest team training not only reduces error but also improves efficiency. If these benefits translate into the physical world, utilizing this type of training in complex surgery could be an easily accessible and effective method of potentially reducing these complications.

One explanation for the association between superior nontechnical skills and reduced intraoperative error could be related to flattening hierarchical gradients, allowing all team members to communicate freely, making operations safer and less error-prone.^35^ Steep hierarchical gradients are thought to lead to more junior team members not challenging questionable decisions made by senior team members.^35^ In both aviation and health care, this has been shown to be harmful.^35,36^ It has been suggested that nurses can feel subservient to doctors in the hierarchy.^35^ Communication has been demonstrated to be more successful under flatter interprofessional hierarchies, leading to improved patient care.^37^ Interprofessional learning and PS together could be an explanation for why the team-trained participants performed more effectively. It is also interesting to note that although the team group in the present study outperformed the solo group in all subdomains of the 3 nontechnical metrics, the difference was marginally more pronounced in the communication subdomains. This suggests that communication is a pivotal factor in the improvement seen.

The concept of interprofessional education (IPE) has evolved recently to introduce this training at an early career stage. Multiple studies support this idea with data suggesting training medical students and technicians together leads to superior nontechnical skills development, better interprofessional relations, and superior patient outcomes.^38,39^ Our study supports this, demonstrating that the virtual world is an ideal place for delivering IPE training without the significant resources and organization constraints associated with more conventional IPE teaching modalities.

Although there is a paucity of data using iVR in a collaborative approach, it has demonstrated success in training surgeons and scrub technicians individually. One of its advantages over other forms of high-fidelity simulation training is that it does not require significant resources or equipment. Headsets can now be purchased for <$500 and are easily transportable.^40^ Furthermore, this training modality is highly efficacious for training both endoscopic and open operations.^41–43^ In a study by Logishetty et al,^19^ 32 surgical residents were trained to perform total hip arthroplasty operations using an AA. Residents improved significantly over the 6-session curriculum reducing the number of assistive prompts received, errors, and procedural times, reaching expert levels by the fourth session. A number of other studies have demonstrated a similar effect in training surgeons, further supporting this concept.^13,43^ More recently this technology has been applied to training scrub technicians. Edwards et al^15^ demonstrated substantial improvement in real-world technical skills scores after a 4-week iVR curriculum for scrub technicians learning revision total knee arthroplasty surgery. The authors also show improvement in confidence and anxiety levels after the training. The present study seems to be the first to combine both roles and the benefits of doing so on teamwork. Future studies could examine adding other roles into the equation to expand this training to the rest of the surgical team.

There are several important factors to consider when applying this data and moving forward. The iVR training program utilized in the present study delivered substantial benefits when used for team training, however was time-intensive. In time-pressured health care systems, implementing a 5-session team iVR curriculum may not be practical, which questions whether these benefits can be obtained over a shorter time period. The authors feel the main reasons for the team groups' superior performance, were related to familiarity, collective PS, and ability to communicate freely without the barriers of work-based hierarchy. This allows teams to work better together and complete the sequence of steps with greater accuracy while being more efficient. However, individual role technical ability was not influenced by team training. There were no differences seen in component orientation (acetabular anteversion and inclination) between groups. This could be because there is little teamwork involved in the surgeon orientating the components. These aspects of the training could potentially be taught separately from a team training focussed intervention, to maximize efficiency.

Another important consideration is whether the benefits seen could be delivered in an alternative medium. The iVR team training allowed participants to gain teamwork skills organically through repetitive practice. However, it may be possible to expedite the development of these skills using targeted teamwork interventions. Classroom or simulation-based team training has been utilized in a number of studies with encouraging results. CRM training, which originates in the aviation industry, focuses on improving nontechnical skills. A study by McCulloch et al^7^ demonstrated how a CRM training program not only improved surgical team nontechnical performance but also improved technical performance and error in 2 commonly performed general surgery procedures. Forse and colleagues found similar benefits by investigating another targeted team training course (TeamSTEPPS). The authors suggested this training led to significant improvements in OR staff teamwork alongside benefits to patient safety with significantly improved mortality and morbidity rates.^44^ It may be possible to expedite the development of teamwork skills with a targeted intervention, potentially delivered in an iVR environment. This method of delivery for targeted nontechnical skills training has yet to be investigated and forms an interesting area for future research.

This study has several limitations. First, although baseline scores were similar between groups, the novice scrub technician group was a diverse mix of student nurses, medical students, newly qualified scrub technicians, and anesthetic assistants. Although the primary outcome was related to overall team performance and not role-specific, this may have introduced some bias and limited how these results can be generalized to the wider population of scrub technicians. In addition, although randomized, when comparing team and solo surgeon groups, those in the team group had marginally more years of residency experience. Although this was not statistically significant, this group also had higher baseline knowledge scores which could have biased the final assessment results. To mitigate a preexisting knowledge discrepancy, we provided an introductory presentation to all participants; however, we did not repeat the baseline assessment to ensure knowledge parity had been achieved. Second, the real-world assessment was conducted in a simulated setting on a high-fidelity model and focused on one operation. This means we cannot comment on how this performance would translate into a real operating room or across different surgical disciplines. Third, although the iVR training modules were identical between solo and team-trained groups, the solo participants were provided with a perfect avatar playing the counterpart role. This could have introduced some bias in their ability to retain information. Finally, as this was a simulated study, the true impact of this intervention on patient safety in the real-world is as yet unknown; this could form an interesting area for future research.

## CONCLUSIONS

Collaborative surgical team iVR training led to the development of superior nontechnical skills alongside more efficient and less error-prone surgery. This multidisciplinary approach using iVR could be feasibly implemented for surgical teams globally, and has the potential to lead to safer and more efficient surgery.

## Supplementary Material
