# Supplementary material for: Collaborative Team Training in Virtual Reality is Superior to Individual Learning For Performing Complex Open Surgery: A Randomized Controlled Trial
Source: Ann Surg. 2023 Aug 28;278(6):850–7. doi: 10.1097/SLA.0000000000006079 (PMC10631503; doi:10.1097/SLA.0000000000006079)
Supplement: Supplementary file 1 [file sla-278-00850-s001.docx]

**Supplementary Table 1.** Inter-observer reliability for all video measurements expressed as the ICC*

| Measurement | Inter-observer Reliability |
| --- | --- |
|  |  |
| NOTSS | 0.92 (0.79-0.97) |
| NOTECHS II | 0.94 (0.84-0.98) |
| SPLINTS | 0.92 (0.80-0.97) |
| Errors | 0.99 (0.98-1.00) |

NOTSS, Non-operative technical skills for surgeons score; NOTECHs, Non-technical skills score ;SPLINTS, Scrub practitioners list of intraoperative non-technical skills ; ICC, Intraclass correlation coefficient

*Intraclass correlation co-efficient, two-way mixed effects model, consistency of agreement, stated as ICC with 95% confidence interval in parentheses

| **Supplementary Table 2.**  Task specific checklist results | | | | |
| --- | --- | --- | --- | --- |
| **Stage** |  | **Step** | **Percent correct** | |
|  |  |  | Team | Solo |
| **Incision and Initial Exposure** | 1 | Palpate/mentions the landmarks (ASIS, greater trochanter, lateral patella) | 100 | 100 |
|  | 2 | Incise the skin in correct orientation | 100 | 100 |
|  | 3 | Incise the fascia | 100 | 100 |
|  | 4 | Pick up Allis clamp to lift the skin and fascia | 95 | 70 |
| **Exposure** | 5 | Pick up Hibbs retractor (retracting skin and fascia) | 90 | 90 |
|  | 6 | Insert Cobra (retracting TFL) | 100 | 65 |
|  | 7 | Readjust Hibbs to reflect medially | 65 | 45 |
|  | 8 | Cobb elevator (push off reflected head of rectus) | 80 | 55 |
|  | 9 | Remove Hibbs | 65 | 25 |
|  | 10 | Insert Curved Hohmann (replace Hibbs) | 60 | 10 |
|  | 11 | Pick up Hibbs to move skin and TFL away | 65 | 40 |
|  | 12 | Diathermy – coagulate vessels (LFCA) | 95 | 95 |
| **Capsular exposure** | 13 | Use Knife (capsulotomy) – T-shaped incision | 100 | 100 |
|  | 14 | Remove Hohmann | 65 | 5 |
|  | 15 | Insert 2^nd^ Cobra around the inferior distal neck of the femur (replacing curved Hohmann) | 50 | 35 |
|  | 16 | Remove Hibbs | 80 | 30 |
|  | 17 | Readjust original cobra and put it around superior and lateral part of femur | 70 | 30 |
| **Dislocation** | 18* | Surgeon adds fine traction | 70 | 35 |
|  | 19 | Use skid to loosen head | 80 | 35 |
|  | 20 | Remove skid | 80 | 35 |
|  | 21* | Surgeon removes fine traction and externally rotates | 60 | 40 |
| **Femoral head resection** | 22 | Drill into femoral head | 100 | 95 |
|  | 23 | Attach T-handle to corkscrew | 100 | 90 |
|  | 24* | Dislocates the hip | 55 | 40 |
|  | 25 | Use Curved Hohmann to put tension on infra-medial capsule | 75 | 50 |
|  | 26 | Use Diathermy release inferomedial capsule off neck | 45 | 15 |
|  | 27 | Remove Curved Hohmann | 85 | 25 |
|  | 28* | Relocates the hip | 50 | 0 |
|  | 29 | Insert Hibbs retractor | 40 | 30 |
|  | 30 | Use oscillating saw for neck resection | 100 | 95 |
|  | 31 | Mallet and osteotome to complete neck osteotomy | 90 | 55 |
|  | 32 | Remove T handle with femoral head | 100 | 85 |
| **Prepare acetabulum** | 33 | Removes retractors | 60 | 45 |
|  | 34* | Maximal external rotation | 50 | 25 |
|  | 35 | Insert Curved Hohmann anteriorly on acetabular rim | 80 | 45 |
|  | 36 | Insert Cobra to reflect capsule inferiorly and push femoral neck posteriorly | 90 | 80 |
|  | 37 | Use scalpel to cut resect labrum | 100 | 65 |
| **Acetabular reaming** | 38* | Ream acetabulum with 48mm reamer | 100 | 100 |
|  | 39 | Ream acetabulum with 50mm reamer | 100 | 100 |
|  | 40 | Ream acetabulum with 52mm reamer | 100 | 100 |
| **Cup insertion** | 41 | Uses mounted cup impactor with correct cup size | 100 | 95 |
|  | 42 | Positions and impacts cup with mallet | 100 | 95 |
|  | 43 | Insert acetabulum liner by hand | 100 | 100 |
|  | 44 | Uses correct impactor to impact liner with mallet | 100 | 85 |
|  | 45 | Remove cobra | 100 | 85 |
|  | 46 | Remove curved Hohmann | 100 | 90 |
|  | 47* | Neutral position leg | 95 | 70 |
| **Femoral preparation** | 48* | Insert Femoral hook support **before** moving traction table | 100 | 70 |
|  | 49* | External rotation, extension, adduction - needs to have all 3 movements | 90 | 50 |
|  | 50 | Insert curved Hohmann | 80 | 80 |
|  | 51 | Insert 90-degree curved Hohmann under greater trochanter | 85 | 50 |
|  | 52 | Use diathermy to perform ischio-femoral ligament release | 55 | 25 |
|  | 53 | Use rongeur or box chisel/osteotome to make entry into femoral canal (posterolaterally) | 100 | 100 |
| **Broaching** | 54 | Mounts broaches in correct order of sizes, starting with size 8 on correct offset handle for a left hip | 100 | 100 |
|  | 55 | Uses mallet to impact broach inwards | 100 | 100 |
|  | 56 | Remove broach using mallet | 95 | 100 |
|  | 57 | Repeat for each size 9->10->11->12, repeats sequentially, starting with smallest size going upwards | 100 | 95 |
| **Femoral trialling** | 58 | Insert standard offset neck | 100 | 100 |
|  | 59 | Insert trial femoral head size 32mm (green) | 90 | 75 |
|  | 60 | Remove 90-degree Hohmann | 100 | 100 |
|  | 61 | Remove Curved Hohmann | 90 | 95 |
|  | 62 | Remove femoral hook support | 100 | 80 |
|  | 63* | Neutral position leg | 100 | 95 |
|  | 64* | Insert Femoral hook before movement | 80 | 45 |
|  | 65 | *External rotation, extension, adduction* | 85 | 70 |
|  | 66 | Insert Curved Hohmann | 80 | 85 |
|  | 67 | Insert 90-degree Hohmann | 90 | 60 |
|  | 68 | Remove trial head | 100 | 100 |
|  | 69 | Remove offset neck | 100 | 100 |
|  | 70 | Pick up broach handle and mallet | 90 | 100 |
|  | 71 | Remove broach using handle and mallet | 90 | 100 |
| **Final implantation** | 72 | Insert femoral stem (size 12) | 100 | 100 |
|  | 73 | Use Stem inserter& mallet to place femoral stem | 100 | 100 |
|  | 74 | Attach Femoral head (32mm) | 100 | 100 |
|  | 75 | Uses correct (femoral head) impactor and mallet to put femoral head in place | 100 | 90 |
|  | 76 | Remove 90-degrees Hohmann | 90 | 95 |
|  | 77 | Remove curved Hohmann | 90 | 95 |
|  | 78 | Remove femoral hook | 100 | 95 |
|  | 79 | Neutral position leg to reduce | 100 | 100 |
| **Clean up** | 80 | Replaces instruments in correct boxes | 100 | 90 |
| **Total** |  |  | 87.0625 | 71.75 |
